# Targeting Alternative Sites on the Androgen Receptor to Treat Castration-Resistant Prostate Cancer

**DOI:** 10.3390/ijms140612496

**Published:** 2013-06-14

**Authors:** Nada Lallous, Kush Dalal, Artem Cherkasov, Paul S. Rennie

**Affiliations:** Vancouver Prostate Centre, University of British Columbia, 2660 Oak Street, Vancouver, BC V6H 3Z6, Canada; E-Mails: nlallous@prostatecentre.com (N.L.); kdalal@prostatecentre.com (K.D.); acherkasov@prostatecentre.com (A.C.)

**Keywords:** androgen receptor, prostate cancer, castration resistance, anti-androgens, protein structure, structure-based drug design

## Abstract

Recurrent, metastatic prostate cancer continues to be a leading cause of cancer-death in men. The androgen receptor (AR) is a modular, ligand-inducible transcription factor that regulates the expression of genes that can drive the progression of this disease, and as a consequence, this receptor is a key therapeutic target for controlling prostate cancer. The current drugs designed to directly inhibit the AR are called anti-androgens, and all act by competing with androgens for binding to the androgen/ligand binding site. Unfortunately, with the inevitable progression of the cancer to castration resistance, many of these drugs become ineffective. However, there are numerous other regulatory sites on this protein that have not been exploited therapeutically. The regulation of AR activity involves a cascade of complex interactions with numerous chaperones, co-factors and co-regulatory proteins, leading ultimately to direct binding of AR dimers to specific DNA androgen response elements within the promoter and enhancers of androgen-regulated genes. As part of the family of nuclear receptors, the AR is organized into modular structural and functional domains with specialized roles in facilitating their inter-molecular interactions. These regions of the AR present attractive, yet largely unexploited, drug target sites for reducing or eliminating androgen signaling in prostate cancers. The design of small molecule inhibitors targeting these specific AR domains is only now being realized and is the culmination of decades of work, including crystallographic and biochemistry approaches to map the shape and accessibility of the AR surfaces and cavities. Here, we review the structure of the AR protein and describe recent advancements in inhibiting its activity with small molecules specifically designed to target areas distinct from the receptor’s androgen binding site. It is anticipated that these new classes of anti-AR drugs will provide an additional arsenal to treat castration-resistant prostate cancer.

## 1. Introduction

Prostate cancer (PCa) is predicted to be the leading cause of cancer-related death in men over the next decade [1]. In its early stages and when localized to the prostate, this cancer can usually be cured by surgery or radiation therapy. However, for advanced, metastatic or recurrent disease, alternative systemic treatments are required. In this regard, the androgen receptor (AR), a ligand-inducible transcription factor, is considered to be central for PCa development, growth and metastasis [2,3].

The AR belongs to the steroid hormone receptor subfamily of the nuclear receptor superfamily. The human AR is coded by a gene located on the chromosome at Xq11-12 and is composed of 919 amino acids. Similar to other nuclear receptors, the AR is organized into three distinct domains: An *N*-terminal domain (NTD), followed by a DNA binding (DBD) and a *C*-terminal ligand binding domain (LBD) [4,5]. Within this family of receptors, the AR shares most of the sequence similarity with progesterone, glucocorticoid and estrogen receptors [6,7]. Steroidal ligands, principally testosterone and dihydrotestosterone (DHT), bind to a ligand binding pocket on the LBD, called the androgen binding site (ABS), to initiate an activation cascade that results in the transcriptional activation of genes that promote PCa cell viability and growth (for review, see [8]). Accordingly, treatment of advanced prostate cancers usually involves some form of surgical or chemical castration to lower the level of circulating androgens and, thereby, to prevent AR transcriptional activity [9]. In addition, to maximize androgen blockade, PCa patients are often treated with drugs called anti-androgens, which compete with naturally occurring androgens for the receptor’s androgen-binding site. Unfortunately, most of these cancers eventually progress to a castration-resistant state, where they no longer respond to androgen deprivation or anti-androgen treatments.

In an attempt to overcome resistance to conventional anti-androgens, computational modeling and high throughput screening techniques have been used to identify small molecules that specifically target functional surface sites of the AR. Several groups have employed this approach to systematically and iteratively optimize small molecules for high-affinity binding to the AR and its effective inhibition [10–15]. The development of such inhibitors has been made possible by investigating the three-dimensional structure of the AR and its co-factors by means of X-ray crystallography, site-directed mutagenesis and biophysical measurements probing the AR’s conformational dynamics and interaction with ligands.

It is important to note that rational, structure-based drug design has been used extensively to develop inhibitors for a number of other nuclear receptors, including estrogen- and progesterone receptors (ER and PR, respectively), and such efforts have resulted in new anti-cancer drugs (for review, see [16]). These successes have motivated a rational drug discovery approach, guided by protein structure and biochemical experiments, to study protein-ligand interactions of the AR and to develop new types of anti-AR therapies. Herein, we provide a focused review on the structure of the AR and its functional regions/domains, which can be targeted with small molecules specifically designed to interact with these regions.

## 2. Androgen Signaling and AR Function in Early and Advanced Prostate Cancer

Androgen-dependent gene transcription by the AR is reliant on a long upstream signal transduction cascade, which, in turn, is preceded by a chain of reactions of androgenic synthesis. Since mutations in the AR can substantially affect its transcriptional activity, understanding the AR structure and function in the context of PCa progression requires comprehensive consideration of the entire AR signaling pathway. All androgen-dependent signaling pathways are driven by testosterone produced in the testes and, to a lesser extent, the adrenals [17]. In androgen target tissues, such as the prostate, testicular testosterone is reduced into DHT by 5α-reductase—An enzyme that has multiple isoforms in humans, some of which are upregulated in cancer [17,18]. DHT is a 10-fold more potent androgen compared to testosterone and plays a greater role in the progression of prostate enlargement and, ultimately, in tumour development [18].

**Figure 1:**
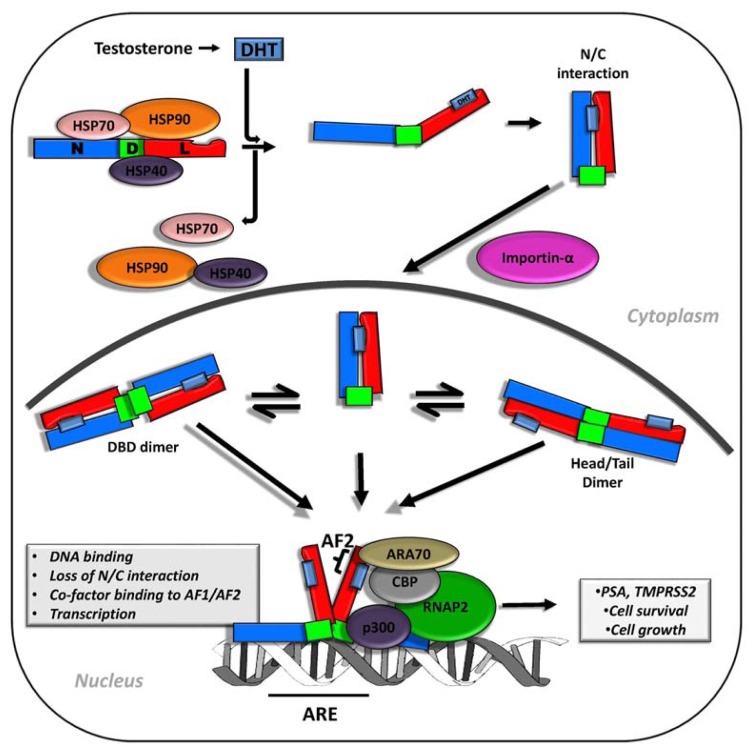
The androgen signaling pathway. The androgen receptor (AR) domains are labeled as: N—*N*-terminal domain (NTD); D—DNA binding (DBD); L—ligand binding domain (LBD). Locally produced dihydrotestosterone (DHT) interacts with the androgen receptor to facilitate release of heat shock proteins (HSP), N/C dimerization and exposure of a nuclear localization signal (NLS) required for interaction with importin-α and nuclear translocation. Inside the nucleus, the AR exists in equilibrium between monomers and dimers. Although it is unclear which form(s) are required for DNA binding, the AR exists as a dimer when bound to androgen response elements (AREs). DNA binding causes the N/C interactions to be lost, allowing the recruitment of transcription initiation and bridging factors, such as CREB binding protein (CBP), the transcription activator p300 and the AR-associated protein 70 (ARA70) to the AR. RNA-Polymerase II (RNAP2) can then transcribe AR-dependent genes.

In the cytoplasm of prostate cells, androgen-free AR is bound by heat shock proteins (HSP) 40, 70 and 90 [19,20], which act as chaperones, maintaining structural integrity of the AR and keeping it in an inactive, ligand-inducible state [19,21]. Upon androgen binding, the AR undergoes conformational changes, which cause a chain of molecular events, including interactions between its N and C termini, release of AR-bound HSP proteins and interaction with co-factors, such as importin-α, which transports proteins across the nuclear pore complex into the nucleus [22–24] (Figure 1). In the classical understanding of the activation of the AR, its nuclear form is phosphorylated by kinases and interacts as a dimer with androgen response elements (AREs) found in the promoters and enhancers of AR-dependent genes. Recent studies suggest that dimerization of the AR only occurs after nuclear translocation and may require prior binding to the DNA [24] (Figure 1). The majority of transcriptional activity of the AR is believed to be conferred primarily through an activation function (AF1) present in its *N*-terminus region (see Section 3) and occurs after recruitment of various factors, including RNA polymerase II and other transcription-initiation proteins [25].

Patients with locally advanced, recurrent or metastatic PCa are usually initially treated with androgen withdrawal therapies, which generally involve either inhibiting androgen synthesis or direct targeting of the AR with anti-androgens, which bind to its androgen binding site. Androgen withdrawal or deprivation consists of reducing the levels of circulating androgens either by surgical castration or by the use of Luteinizing hormone-releasing hormone (LHRH) agonists [26–28]. Although not the subject of this review, a variety of inhibitors that impair androgen synthesis pathways, without direct interaction with the AR, are often used as a second line therapy [29–32]. Among the inhibitors that block key enzymes in the androgen synthesis pathway are ketoconazole, abiraterone, orteronel (TAK-700) and galeterone (TOK-001) [33–38]. Ketoconazole inhibits CYP11 (cholesterol side-chain cleavage enzyme) and CYP17 (17α-hydroxylase/17,20 lyase/17,20 desmolase), members of the cytochrome P450 superfamily [33,34]. Abiraterone, orteronel and galeterone are inhibitors of CYP17 [31,35–38]. Abiraterone is currently the main drug used as a second line therapy to block intracrine production of androgens within prostate tumours [35].

To achieve maximal blockade of androgen signaling, anti-androgens are often used in conjunction with androgen withdrawal therapies [39,40] (discussed in detail in Section 4). Although the exact mechanism of action of these compounds remains to be elucidated, competition for the ligand binding site is thought to be the main mode of action and, thereby, prevents nuclear localization of the AR and its activation of transcription (for recent reviews, see [31,41]). While not yet used clinically, RNA interference, using either short hairpin or small interfering RNA (shRNA and siRNA), has been shown to cause tumour regression in PCa xenograft models [42–45].

Although the above methods are initially effective in blocking the AR signaling, patients eventually develop castration-resistant prostate PCa (CRPC), which is characterized by rising serum prostate specific antigen (PSA) and renewed tumour growth [2,3,46,47], with subsequent poor patient survival [48,49]. Notably, expression of AR-dependent genes, such as PSA and transmembrane protease serine 2 (TMPRSS2), is often reactivated in CRPC, despite low levels of circulating androgens, implying that AR transcriptional activity has become ligand-independent [50]. The recurrence of AR signaling appears to have multiple origins, further complicating the understanding of mechanisms of CRPC occurrence and progression. Hallmark characteristics of CRPC tumours include, but are not limited to: Increased levels of AR or its mRNA [17,51,52]; somatic mutations in the AR sequence (such as T877A) that convert anti-androgen drugs into agonists [51]; upregulation of enzymes responsible for androgen synthesis [53]; direct alteration of the AR gene [54]; and constitutive transcriptional activity of truncated AR splice variants [55–59] (discussed in Section 3). Comprehensive descriptions and summaries on reported mechanisms of AR reactivation in CRPC can be found elsewhere [2,54,60].

## 3. AR Structure and Domain Organization

### 3.1. *N*-Terminus Domain (NTD)

The multifunctional role of the AR is implemented through its modular domain organization. The AR *N*-terminus domain (NTD) corresponds to the first 558 residues and contains the AF1 functional region. The NTD is the least conserved domain amongst all nuclear receptors. Accordingly, the EMBOSS metrics [61] demonstrate that the NTD of human AR shares only 8.4% sequence identity with the human estrogen receptor (ER) NTD, 14.9% identity in the case of the glucocorticoid receptor (GR) and 21.9% with the progesterone receptor (PR). The NTD is characterized by the presence of two large repeats, termed homopolymers: Poly-glutamine and poly-glycine fragments, averaging 21 and 24 residues, respectively. The variation in length of the poly-glutamine has been associated with such diseases as X-linked spinal and bulbar muscular atrophy and prostate cancer [62–65]. Other shorter repeats also exist in this AR region, including two poly-glutamine stretches (five to six residues in length), a poly-alanine (five residues), a poly-proline (eight residues) and two amino-acid repeats with the PSTLSL sequence.

The NTD of the AR contains a number of functionally important regions, such that deletion of portions of the NTD or mutation of some of its key residues (such as I229A/L236A; L251A/L254A, M244A, L246A, V248A) can lead to a decrease of AR transactivation activity [66,67]. Furthermore, two transcriptional activation units (TAU) were mapped onto the AR NTD: TAU-1 (residues 101–370) and TAU-5 (residues 360–485) [67,68]. Importantly, the AR-NTD contains an FXXLF motif at residues 23–27 and a WXXLF motif at residues 433–437 that are essential for the interaction with the AR’s LBD. The corresponding contact (termed N/C interaction) has been shown to be critical for stabilizing the androgen in the ligand binding pocket and for overall AR function [69,70].

The AR NTD also plays an essential role in a number of protein-protein interactions [71,72]. It has been shown that the NTD serves as a binding site for many transcription machinery components, including TFIIF and TFIIH proteins [73–75], co-activators, such as CREB-binding protein (CBP) [76,77], and co-repressors, like SMRT (silencing mediator for retinoic acid and thyroid hormone receptor) [78]. Despite the importance of the NTD for AR function, currently, there is no structural information available for this domain, in part due to intrinsic disordered regions that compromise the stability of the NTD in solution and prevent its crystallization [72].

### 3.2. The DNA Binding Domain (DBD)

**Figure 2:**
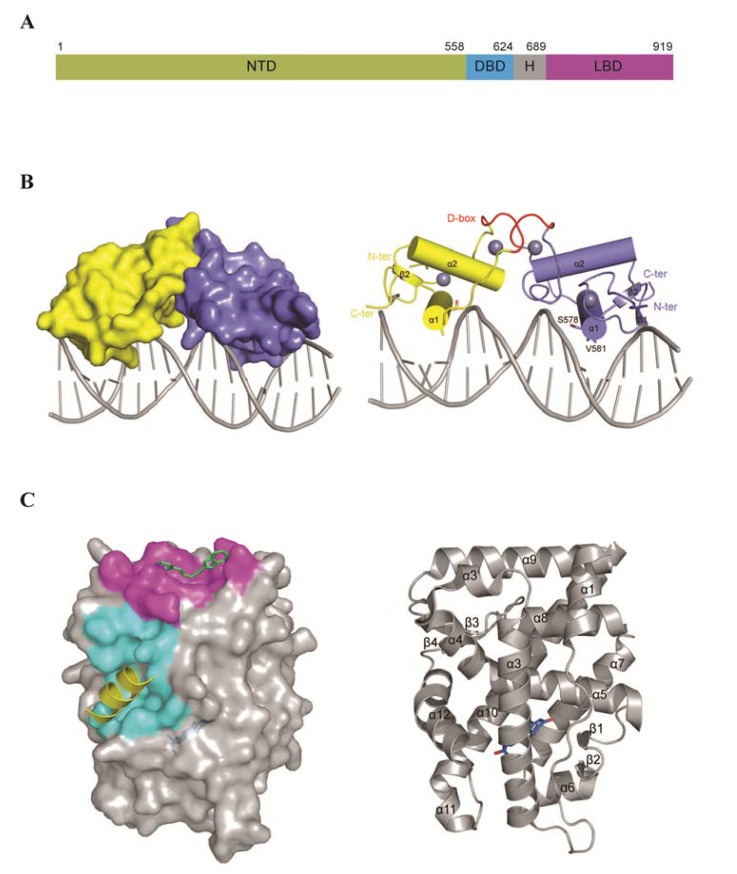
Domain organization and available structures of the androgen receptor. (**A**) Scheme of the domain organization of the AR: NTD (*N*-terminal domain), DBD (DNA binding domain), H (hinge region) and LBD (ligand binding domain). Residue numbers above the scheme delineate the domain boundaries; (**B**) Surface (**left**) and cartoon (**right**) representations of the rat AR-DBD structure. Zinc ions are presented as grey spheres, and the D-box of each DBD monomer is highlighted in red. Residues of the P-box involved in DNA recognition are shown as sticks; (**C**) Surface (**left**) and cartoon (**right**) representations of the human AR-LBD structure: surfaces highlighted in cyan and magenta correspond to AF2 and BF3, respectively. The cartoon representation in yellow corresponds to a coactivator bound to AF2, and the stick representation in green depicts a BF3 small molecule inhibitor. R1881, a synthetic androgen, bound to the androgen binding site, is shown in blue.

The DBD is highly conserved among NRs. In this regard, the AR DBD shows 79.5% identity with the corresponding regions of the PR, 71.2% with the GR and 53.4% with the ER. This 66 amino acid-long domain contains two zinc finger motifs, where each metal ion is coordinated by four cysteine residues. The first motif contains the P box (residues 577–581: GSCKV), which interacts with the major groove of the DNA, while the second zinc finger contains the D box (residues 596–600: ASRND), which plays a role in DBD-mediated AR dimerization (Figure 2B) [79]. The AR-DBD recognizes classical androgen response elements (AREs) on the DNA that are organized as inverted repeats of 5′-AGAACA-3′-like motifs with a three nucleotide spacer (IR3) and selective AREs that are considered direct repeats of 5′-AGAACA-3′-like motifs (DR3) [80–82].

There is only one crystal structure available for the rat AR-DBD, which was resolved in a complex with a DR3 response element (PDB: 1R4I) formed by two hexameric half-sites arranged as a direct repeat separated by three base pairs (CC***AGAACA***TCA***AGAACA***C) [83]. According to the reported crystal structure, the AR-DBD is formed by two short anti-parallel β-strands and two perpendicular α-helices. This organization allows the AR-DBD to bind to the DNA in the form of a “head to head” dimer, where one monomer binds the half-site response element with high affinity and the second binds the other half-site with lower affinity [83].

### 3.3. Hinge Region

The region between the DBD and the LBD (residues 625–689) is flexible and poorly conserved among NRs. The signal responsible for nuclear import is encoded by a bipartite nuclear localization signal (NLS: 617-**RK**CYEAGMTLGAR**KLKKL**-634) formed by two clusters of basic residues belonging to the *C*-terminus of the DBD and the *N*-terminus of the hinge region [84–86]. The cellular localization of the AR is controlled by androgen binding, such that the AR is cytoplasmic in its ligand-free state, but upon binding of androgen to the LBD, it undergoes a conformational change, which exposes the NLS and facilitates its interaction with importin-α, which results in translocation of the activated AR to the nucleus [22,87].

### 3.4. Ligand Binding Domain (LBD)

The LBD is composed of 11 α-helices (numbered 1–12, where helix 2 is missing compared to other NRs), arranged as a three-layered helical sandwich and four β-strands organized in two short sheets (Figure 2C). The androgen binding site of the LBD is formed by residues belonging to β1 and helices 3, 5, 7 and 10. These residues make hydrogen bonds and/or hydrophobic interactions with testosterone and DHT moieties [88]. The LBD surface also contains a hydrophobic cleft, known as the Activation Function 2 region (AF2), formed by residues belonging to helices 3, 3′, 4 and 12 (Figure 2C). This pocket is structurally completed upon androgen binding, due to induced conformational changes in helix 12 [89]. This movement of helix 12 was captured in the estrogen receptor-α crystal structures resolved for both agonistic and antagonistic forms of the receptor [90]. In the case of the AR, this pocket is a binding site for numerous coactivators, including members of the p160 SRC family, such as steroid receptor coactivator-1 (SRC1), TIF2/GRIP1/SRC2 and SRC3 [91]. Additionally, AR AF2 recognizes, with high affinity, the NTD FXXLF motif to form a strong N/C interaction, essential for AR-dependent gene regulation [69,70,92].

In recent years, an additional surface pocket, called binding function 3 (BF3), was discovered by Dr. Robert Fletterick’s group [93]. This site is distinct from the androgen binding and AF2 sites and is believed to allosterically regulate the AF2 [93,94]. The AR BF3 is formed by residues from helices 1, 3′ and 9 and the loop connecting H3 and H3′regions in the LBD. This surface has been reported to be important for FKBP52-dependent AR regulation; however, no direct binding between the two entities has yet been demonstrated [95]. It has been documented that, in addition to known mutations in the androgen binding site, the AF2 and BF3 areas are also associated with PCa and androgen insensitivity syndromes [96]. Given the importance of these sites for AR function and modulation, they clearly represent prospective targets for developing novel PCa treatments.

### 3.5 Splice Variants

**Figure 3:**
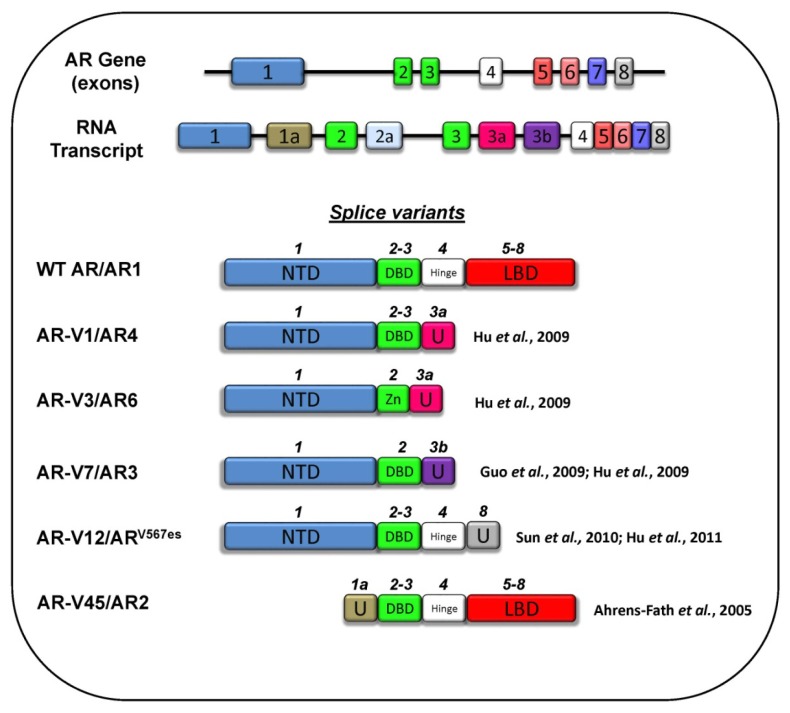
Common splice variants of the AR. The AR gene is organized as eight exons, which form the coding sequence for its different domains: NTD—exon 1; DBD—the first and second zinc finger motifs are encoded by exon 2 and 3, respectively; Hinge—exon 4; LBD—exons 5–8. The RNA transcript can by spliced in several ways to include combinations of the standard exons (numbered 1–8), as well as inclusion of cryptic exons (1a, 2a, 3a, 3b). Shown above each splice variant are the corresponding exon numbers included in spliced mRNA. Cryptic exon inclusion results in unique (U) regions with novel nucleic acid sequences not found in the wild-type (WT) AR. In AR-V3/AR6, the inclusion of exon 2 yields a splice variant bearing only one zinc finger (Zn) and, thus, a truncated DBD.

Alternative splicing of RNA transcripts is a mechanism used by cells to increase the diversity of functions of individual genes. The wild-type full length AR consists of eight exons and introns that can be spliced into a plethora of forms, some lacking entire domains (Figure 3). AR splice variants arise primarily through exon skipping and cryptic exon inclusion, where splicing introduces RNA sequences not normally included in the transcript [97]. Although some splice variants, such as AR-45 [98], are found in normal prostate tissue, variants lacking the LBD have been found to be upregulated in tumours (compared to levels in normal prostate cells) [99–102]. In particular, one such truncated form, AR-V7/V3, has been postulated to be a major androgen-independent driver of AR-regulated gene expression in CRPC [55,99,101].

## 4. Targeting the LBD

Most of the current clinically used non-steroidal anti-androgens, including flutamide, nilutamide and bicalutamide (Casodex), target the androgen binding site of the LBD. These drugs compete with endogenous androgens to inhibit AR transcriptional activity. A major complicating factor with continuous treatment using these drugs is the emergence of AR mutations, which can cause resistance or even convert them into AR agonists [103].

**Figure 4:**
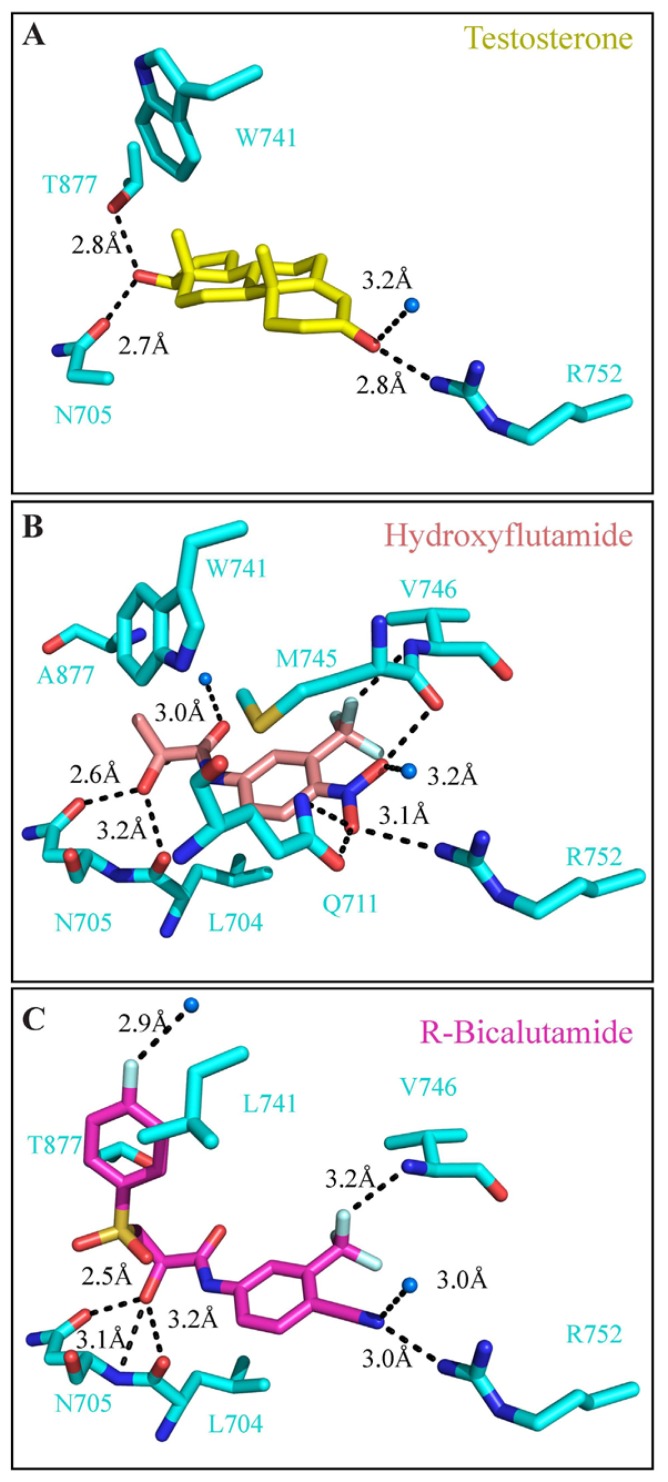
Detailed view of the androgen binding site in complex with (**A**) testosterone, (**B**) hydroxyflutamide and (**C**) R-bicalutamide. The hydrogen bonds and ionic interactions between the protein and the ligand are shown as dashed lines.

Presently, there is no information available on the structure of AR-LBD with bound anti-androgens, due to complications in obtaining a pure LBD/anti-androgen sample suitable for X-ray crystallography. However, different X-ray structures have been solved for the mutated agonist-converting forms of the AR-LBD (such as T877A and W741L) in complex with these drugs [104–107]. The pairwise comparison of available crystal structures has revealed that the overall configuration of the AR-LBD in complex with testosterone (PDB: 2AM9) [88], R-bicalutamide (PDB: 1Z95) [104] or hydroxyflutamide (PDB: 2AX6) [105] is highly conserved. The root mean square deviation (RMSD) values calculated for all the backbone chains in the crystal structures are: 0.334 Ǻ (235 Cα) for *R*-bicalutamide/testosterone and 0.323 Ǻ (239 Cα) for hydroxyflutamide/testosterone. According to the published structure (PDB: 2AM9), the testosterone molecule establishes hydrogen bonds with R752, N705 and T877 residues and forms a bond with one water molecule (Figure 4A). Hydroxyflutamide and *R*-bicalutamide present the same electrostatic interactions with R752 and N705 side chains (Figure 4B,C). The coordinating water molecule is conserved in all three structures. Additionally, both drugs present extra hydrogen bonds with the main chain nitrogen of V746, due to their fluorine group (F2), with the main chain oxygen of L704 and with an additional water molecule.

Of note, the structure of the T877A AR mutant complexed with hydroxyflutamide presents a new conformation of the W741 side chain (Figure 4B) compared to its conformation in the testosterone structure (Figure 4A). This alternative position of W741 is important to allow a water molecule to establish an additional hydrogen bond with a carbonyl group of hydroxyflutamide and also to accommodate two extra methyl groups from the drug. In the structure of *R*-bicalutamide bound to the W741L AR mutant, the B ring of the ligand occupies the location that was previously filled by the indole ring of the mutated tryptophan. The fluorine group on the B ring forms a hydrogen bond with an additional water molecule. This structural information suggests possible clashes between the antagonists and WT configurations of the LBD, supporting eventual conformational changes or partial unfolding of the AR in the presence of these anti-androgens. This might be due to helix 12 displacement, as was seen with the estrogen receptor–α and the glucocorticoid receptor antagonist structures [108–110].

The anti-androgen enzalutamide (MDV-3100) was recently approved by the FDA for treating metastatic PCa and CRPC. This drug has an IC_50_ of 21 nM, which is an eight-fold higher affinity for the AR compared to bicalutamide (IC_50_ = 160 nM) [39], and additionally inhibits AR nuclear translocation [39,111,112]. No experimental structural information is available on how exactly enzalutamide interacts with the AR, although crystal structures do exist for the AR-LBD in complex with enzalutamide-like agonists (PDB: 3V49 and 3V4A) [113]. Due to the specificity of protein-ligand interactions, more insight into enzalutamide binding to the AR is needed in order to better understand its antagonistic action.

A more recent experimental anti-androgen, called ARN-509, is structurally similar to enzalutamide and is currently in phase I/II clinical trials [114]. This compound also impairs AR nuclear translocation and DNA recognition by the AR-DBD. Although ARN-509 has the same *in vitro* behavior as enzalutamide, it shows three-fold better efficacy in CRPC mouse models and has fewer known side-effects [41,114].

Recent advances in the area of rational and computer-aided drug design have resulted in the development of a number of other candidate anti-androgens targeting the androgen-binding site, including compounds, such as 6-(3,4-dihydro-1*H*-isoquinolin-2-yl)-*N*-(6-methylpyridin-2-yl)nicotinamide (DIMN) [12], its derivatives, termed 7AU and 7BB [115], and 8-(propan-2-yl)-5,6-dihydro-4*H*-pyrazino[3,2,1-jk]carbazole (MEL-3) [116], all showing promising *in vitro* and *in vivo* activities and currently undergoing various stages of pre-clinical development.

Due to limitations associated with targeting the androgen binding site, finding alternative target areas on the AR has become a major investigational focus. Although the NTD and DBD parts of the protein represent attractive targeting options, (see Section 5 and 6), alternative surface sites on the LBD itself, including the already mentioned AF2 and BF3 functional pockets, remain to be exploited. It is likely that compounds acting on these LBD surfaces would target the receptor by a completely different mechanism compared to conventional anti-androgens, possibly by directly disrupting coactivator recruitment.

Recent studies of compounds designed to bind alternative sites on the LBD surface have shown promising levels of *in vitro* inhibition of AR transcriptional activity. In a previous study, Estebanez-Perpina *et al.* [93] used a fluorescence polarization assay to screen for compounds that bind to the AR AF2 area and that also inhibit its interaction with a SRC2–3 activator peptide. In their screen, they found that two known drugs, triac and flufenamic acid, were able to bind to the AF2 site and block AR transcriptional activity in a cell-based assay (with luciferase reporter). Surprisingly, some of the identified AF2 binding compounds were shown to also bind to the neighboring BF3 surface. The corresponding X-ray structures (PDBs: 2PIX, 2PIU) also highlighted ligand-induced allosteric changes in residues R840, K717 and M734, which form the AF2 site. These changes seemed to be sufficient to disrupt coactivator binding to the AR [94,117].

Our laboratory is also working on targeting the AF2 and BF3 surfaces of the AR in order to develop a new class of inhibitors that can be used alternatively or complementarily to current PCa and CRPC therapies. Using an *in silico* drug discovery approach integrated with biological validation, we identified several potent small molecule inhibitors selectively targeting the AR AF2 and the BF3 sites [10,13,118]. These compounds were able to inhibit AR activity with corresponding IC_50_ values in the sub-micromolar and nanomolar ranges. Furthermore, these proto-drugs also demonstrated inhibition of endogenous PSA expression and secretion in LNCaP PCa cells, as well as effective cell killing in MTS assays. Importantly, the compounds were effective in inhibiting AR activity and causing cell death in enzalutamide-resistant PCa cells [13]. Owing to their distinct AR target sites, there was no apparent cross-resistance observed for the anti-AF2 and anti-BF3 drug prototypes. To validate the on-target binding of these compounds, X-ray structures were resolved with some of the developed inhibitors bound to the AR AF2 or the BF3 sites (PDBs: 2YLP, 2YLO, 2YHD, 4HLW). It is worth noting that AR AF2 and BF3 pockets are highly conserved among NRs, with up to 58% sequence identity for some family members [117]. Therefore, the cross-reactivity of AF2 and BF3 inhibitors with other NRs should be verified in the future, at least with the most efficient compounds.

## 5. Targeting the *N*-Terminal Domain

The identification of small molecules capable of binding to the AR-NTD has proven to be an elusive goal, given that no structural information is available for this domain. However, since both ligand-dependent and -independent transcriptional activity of the AR is attributed to its *N*-terminal Tau1 and Tau5 regions (see section 3.1), the NTD remains a very attractive drug target for treating both early stage PCa and CRPC. To date, three classes of NTD inhibitors have been reported in the literature: (1) cyclical peptides, termed sintokamides that were isolated from marine sponges; (2) decoy peptides containing the AR-NTD sequence; and (3) a small molecule referred to as EPI-001, which is presumed to bind to the AR-NTD and block activating protein-protein interactions of the AR [119].

The peptide-based AR-NTD inhibitor was originally isolated by applying a high-throughput screening approach with a library of natural marine extracts [120]. This led to the discovery of sintokamides, small peptides with varying degrees of chlorination, which were isolated from the marine sponge *Dysidea* sp. [120]. One such peptide variant, sintokamide A, demonstrated inhibition of the growth of the androgen-dependent LNCaP PCa cell line. In addition, sintokamides showed inhibition of transcriptional activity of the NTD fragment of the AR fused to a Gal4 DBD domain (using a luciferase reporter and Gal4 promoter sequence). Since the Gal4-NTD construct has no ligand binding domain, it was suggested that sintokamides are also effective in supressing the AR activity under androgen-independent conditions. A more recent high-throughput screen of extracts from the marine sponge *Niphates digitalis* has yielded additional candidate compounds that also antagonize AR transcriptional activity [121]. Furthermore, it has been proposed that one such compound, niphatenone B alkynyl ether, may covalently bind to the AF1 region of the NTD [121].

As an alternative approach, a decoy peptide comprising the entire AR-NTD sequence (from amino acid 1 to 538) has been shown to inhibit the transcriptional activity of the full length AR (detected with a PSA reporter construct) [122]. The mechanism of action of the AR_1–538_ peptide remains unclear, but it likely competes with known AR cofactors for NTD binding. It has also been shown that AR_1–538_ peptide is effective in both ligand-dependent and -independent conditions. Conceivably, such a decoy peptide may alternatively bind directly to the AF2 of the full length AR, thereby preventing the N/C self-activating interaction facilitated by the FXXLF sequence motif [123,124], which is contained within the first 30 residues of the NTD [24,123,125].

In another study, Andersen *et al.* [126] reported on the high throughput screening discovery of EPI-001, a small molecule candidate inhibitor for the AR-NTD. This molecule, being a close analog of bisphenol A diglycidic ether, could block transcription of a PSA reporter construct in LNCaP cells. In addition, EPI-001 showed similar activity in 22rv1 cells, which contain both full length AR and the AR-V7 splice variant (Figure 3). Using a PSA reporter construct, EPI-001 was shown to also inhibit the transcriptional activity of a truncated form of the AR lacking the LBD domain (AR_1–653_). Together, these results suggest that EPI-001 has the capability to directly target the NTD of the AR splice variants. Importantly, the activity of progesterone receptors and glucocorticoid receptors was not affected by EPI-001, which illustrates its specificity for the AR. However, in the absence of the AR-NTD crystal structure, it is unclear how EPI-001 binds to the AR. Nevertheless, intrinsic fluorescence of tyrosine and tryptophan residues within an AF1 peptide could be modulated by this compound, suggesting direct binding to the NTD. Whether EPI-001 binds to the AF1 region within the context of the full length AR remains to be determined. Some support for this interaction was shown by co-immunoprecipitation experiments with the AR, where EPI-001 caused a modest (21%) reduction of the pull-down of CBP, a co-factor known to bind to the AR AF1. The inhibitory effect of EPI-001 *in vivo* was validated by showing a volume reduction in CRPC tumour xenografts in mice. Currently, EPI-001 is the best characterized compound that appears to target and inhibit the activity of the AR-NTD and, therefore, is a good candidate drug for treating CRPC.

## 6. Targeting the DNA Binding Domain

To date, there has been little progress in the development of AR inhibitors that target the AR-DBD domain [16]. Factors limiting progress in this area of research include high sequence identity between the DBDs of all steroid receptors, which could cause specificity problems for the corresponding AR-DBD-directed compounds. Nevertheless, a recent report using high throughput screening of ~160,000 molecules identified one relatively AR-specific inhibitor, 1-(3-(2-chlorophenoxy) propyl)-1*H*-indole-3-carbonitrile, which could potentially interact with the DNA binding domain of the receptor [127]. Chromatin immunoprecipitation experiments indicated that this compound interferes with the binding between the AR and the PSA or TMPRSS2 gene promoters [127]. Western blot analysis also demonstrated that this candidate AR-DBD inhibitor did not affect the level of AR expression. Furthermore, a fluorophore-tagged antibody revealed that the AR was predominantly localized to the nucleus of cells treated with the compound [127]. As a result, it was concluded that 1-(3-(2-chlorophenoxy) propyl)-1*H*-indole-3-carbonitrile likely targets the AR-DBD in such a way that it modulates AR interaction with DNA. Importantly, using a luciferase reporter assay to measure transcriptional activity, this compound did not affect the GR and showed a modest effect on ERα, but only at high concentration [127]. However, additional experiments are needed to demonstrate the direct interaction between this compound and purified AR-DBD.

Development of chemical agents that bind to androgen response element (ARE) sequences to prevent the AR docking onto the DNA has also shown considerable promise. In particular, pyrrole-imidazole (Py-Im) polyamides, sequential arrangements of *N*-methylpyrrole and *N*-methylimidazole carboxamide monomers, may be useful in this regard [128]. Typically, polyamide strands are linked by a γ-amino acid at one end to generate a hairpin or at both ends to create a cyclical structure. These compounds can specifically interact with the minor groove of DNA and have the ability to bind to G-C or A-T base pairs (depending on the polyamide sequence). In addition, polyamides can allosterically affect the conformation of the double stranded DNA and, thus, prevent protein-DNA interactions [128].

In a recent study, Dervan and coworkers [129] demonstrated that hairpin polyamides could bind to the PSA promoter and downregulate its mRNA expression with the same efficiency as the anti-androgen bicalutamide. Building on these results, Chenowith *et al.* [130] showed that cyclical polyamides have improved binding to the AREs and can effectively inhibit PSA expression. In mice, polyamides demonstrate favourable cell permeability, high solubility, long half-lives and low toxicity. Recently, a polyamide agent directed against the 5′-NGNNCN-3′ sequence of the ARE was tested in mice and shown to lower circulating PSA levels, to activate the p53 gene and to cause apoptotic cell death in LNCaP xenografts [14]. In the presence of this polyamide, the occupancy of RNA polymerase II (RNAP2) onto AR-dependent genes was also reduced, as measured by CHIP-seq analysis. Moreover, Western blots of LNCaP cells treated with this polyamide exhibited marked degradation of the RPB1 subunit, a known elongation factor for RNAP2. Considering all the evidence, Yang *et al.* [14] suggested that polyamides may weaken the recruitment of RNAP2 to AR specific promoters, resulting in the compromised polymerase elongation. The applicability of polyamides for treating CRPC will depend on the ability of these compounds to simultaneously target multiple AR-dependent genes that have a role in disease progression. Whether a mixture of polyamides designed to specifically target different AREs will be effective in downregulating AR transcriptional activity in CRPCs remains to be seen.

## 7. Future Outlook

Understanding the three-dimensional structure and function of AR domains has played a major role in the development of inhibitors against the receptor’s transcriptional activity. Whereas most small molecules have been designed to compete with DHT for the androgen binding site in the AR-LBD, additional distinct, yet functionally significant pockets in other regions of the protein, including the BF3 and AF2 surface sites, as well as DBD and NTD domains, should also be targeted to deal with CRPC.

While AR-NTD inhibitors show promise in blocking AR transcriptional activity and treating CRPC tumours driven by AR variants, more detailed information on the structure of the AR-NTD is necessary in order to understand the molecular mechanism of action of small molecule inhibitors, such as EPI-001, as well as peptide molecules, which appear to interact with the AF1 region. At the very least, site-directed mutagenesis of amino-acid residues in the AF1 region should be performed when testing NTD inhibitors to provide evidence that they indeed interact directly at the expected site on the AR.

The AR-DBD may allow for a more rational approach to drug design, given that its crystal structure is already known [83]. In particular, exposed regions at the DBD dimerization (D-box) and DNA binding (P-box) interfaces (Figure 2A) could potentially be targeted by small molecule inhibitors. This will likely require accurate *in silico* modelling coupled with high throughput screening of small molecules, as well as assessment of their inhibitory effect on AR transcriptional activity using reporter assays. Mutagenesis of key amino-acids in the D-box and P-box weakens the dimerization and transcription factor activity of the AR [24] and clearly shows the importance of these surface exposed regions. Thus, AR-DBD-interfering inhibitors should have the potential advantage of preventing all AR transcriptional activity, including the truncated constitutively active ones, rather than targeting a subset of androgen-dependent genes, such as that achieved using polyamide treatments. It is important to ensure that small molecules directed against the AR-DBD should not cross-react with the DBDs of other nuclear receptors. Presumably, this could be achieved by a detailed comparison of the spectrum of DBD surfaces, while rationally designing the corresponding lead inhibitors. More structural information of the AR-DBD complex with different AREs is now required to validate the underlying mechanisms of DNA binding and dimerization.

The search for new small molecule inhibitors that target alternative pocket sites and surface exposed regions of the AR has intensified considerably in recent years. It has become clear that androgen deprivation and other currently used hormone therapies have inherent limitations, partly due to recent discoveries pertaining to AR splice variants lacking an LBD (e.g., AR-V7) that are implicated in the reactivation of AR signaling in CRPC. Small molecules that target sites on the AR distinct from the androgen binding site are expected to have completely different mechanisms of action for inhibiting AR signaling than conventional anti-androgens and are, therefore, less likely to be cross resistant when used as a second line of therapy. Furthermore, there is a strong potential for these other novel forms of AR inhibitors to be used synergistically with potent clinically used anti-androgens, such as enzalutamide, to achieve a more substantial anti-tumour response. We anticipate that a complete picture of the AR structure, using crystallographic, NMR and biochemical methods, will lead to significant drug design breakthroughs in the coming years.
